# Unveiling the Therapeutic Potential of Vitamin-E in Preventing Inflammation and Stromal Congestion in Alcoholic Liver Injury

**DOI:** 10.7759/cureus.54931

**Published:** 2024-02-26

**Authors:** Noman Ullah Wazir, Tania Khattak, Usama Khan, Saman Hussain, Syed Mohammad Tahir Shah, Muhammad Haris, Sobia Haris, Farah Deeba

**Affiliations:** 1 Department of Anatomy, Peshawar Medical College, Peshawar, PAK; 2 Department of Pathology, Nowshera Medical College, Nowshera, PAK; 3 Department of Internal Medicine, Nowshera Medical College, Nowshera, PAK; 4 Department of Pathology, Northwest School of Medicine, Peshawar, PAK; 5 Department of Surgical Specialties, Type-D Hospital Shahbaz Garhi, Mardan, PAK; 6 Department of Anatomy, Nowshera Medical College, Nowshera, PAK; 7 Department of Healthcare & Hospital Management, BRAINS Institute, Peshawar, PAK; 8 Department of Anatomy, Institute of Basic Medical Sciences, Khyber Medical University, Peshawar, PAK; 9 Department of Medical Education, Nowshera Medical College, Nowshera, PAK; 10 Department of Health Research, Nowshera Medical College, Nowshera, PAK

**Keywords:** central vein, hepatic sinusoids, vitamin e, portal inflammation, alcoholic liver injury

## Abstract

Objectives: The objective of the research was to investigate and assess how effective Vitamin E is in preventing or reducing liver inflammation and stromal congestion associated with alcoholic liver injury.

Study design: This is a laboratory-based experimental study.

Methodology: A total of 18 domestic rabbits were divided into groups A, B, and C. Group A was the control group and treated with normal saline as a placebo. Groups B and C were given 30% ethanol in a daily dose of 30 ml/kg/day. Additionally, group C was treated with vitamin E at 50 mg/kg/day. All three groups were sub-divided into two sub-groups I and II on the basis of experimental duration of eight weeks and four weeks respectively. The subgroups with eight weeks of experimental time duration were categorized as "category E8" and subgroups with an experimental duration of four weeks were categorized as "category E4". Liver tissue samples from each animal were subjected to staining using hematoxylin and eosin (H&E) stain for histological staining in order to assess portal inflammation and to measure the sizes of hepatic sinusoids and central veins to evaluate hepatic congestion.

Results: A statistically significant variance was observed in the size of central veins, hepatic sinusoids, and invasion of inflammatory cells in portal areas across and between the groups within categories E4 and E8. Animals treated with vitamin E exhibited lower invasion of inflammatory cells and larger central veins and sinusoids compared to those not treated with vitamin E.

Conclusion: Vitamin E may have a significant role in reducing or limiting the infiltration of inflammatory cells and could help prevent hepatic congestion in cases of alcoholic liver injury.

## Introduction

Alcoholic liver damage continues to pose a substantial global health challenge, involving various pathological stages ranging from fat accumulation in the liver (steatosis) to inflammation (steatohepatitis), fibrosis, and eventual cirrhosis [1]. Alongside the harm to liver cells, alcoholic liver disease (ALD) is marked by significant infiltration of inflammatory cells and congestion in the liver's supportive tissue (stroma), both of which significantly contribute to the advancement of the disease [2]. Various types of immune cells, such as neutrophils, macrophages, T cells, and natural killer cells, invade the liver and mostly populate the portal triad areas in the liver when it's injured by alcohol [3]. These cells unleash inflammatory molecules like cytokines, chemokines, and reactive oxygen species, which worsen liver inflammation and damage. When certain immune cell populations lose their balance, it intensifies the inflammatory reaction, accelerating the progression of ALD [4]. At the same time, the metabolism of alcohol produces reactive oxygen species (ROS), which subsequently cause oxidative harm and congestion of the liver stroma [5].

Apart from the infiltration of inflammatory cells, alcoholic liver injury presents with liver stromal congestion, where extracellular matrix components such as collagen and fibronectin accumulate [6]. This congestion disrupts hepatic architecture, leading to impaired liver function and facilitating fibrogenesis [7]. Moreover, sinusoidal endothelial dysfunction and compression of both sinusoidal and central veins alter blood flow dynamics, exacerbating stromal congestion and perpetuating liver injury in ALD [8]. Focusing on reducing the infiltration of inflammatory cells and alleviating liver stromal congestion presents a hopeful avenue for treating ALD [9]. Approaches targeting the regulation of immune cell movement, suppression of inflammatory signaling pathways, and reduction of stellate cell activation show promise in improving liver damage and fibrosis in patients with ALD.

Vitamin E, a powerful antioxidant soluble in lipids, has attracted attention due to its ability to combat oxidative stress and inflammation in diverse pathological contexts, including ALD [10]. The primary form of vitamin E, α-tocopherol, functions as an antioxidant by scavenging free radicals and bolstering the stability of cellular membranes, thus shielding against lipid peroxidation and consequent tissue harm [11,12]. Furthermore, vitamin E has been associated with regulating immune responses and cytokine production, suggesting a potential role in mitigating alcohol-induced hepatic inflammation. Renowned for its antioxidant properties, vitamin E can potentially counteract detrimental free radicals within the body [13]. This study investigates the potential protective effects of vitamin E against the invasion of inflammatory cells and stromal congestion in the liver during episodes of alcoholic liver injury.

## Materials and methods

The investigation took place at the Department of Anatomy, Peshawar Medical College in Peshawar, Pakistan. Approval was granted by the Institutional Review Board (IRB) of Prime Foundation (protocol number Prime/IRB/2017-547). Eighteen healthy adult rabbits of exclusively male gender, belonging to the domestic breed (*Oryctolagus cuniculus*) were selected for the study. These rabbits, approximately one year old, weighed between 1 and 1.5 kg. They were housed in carefully constructed iron cages with a natural soil floor, maintained under standardized environmental conditions. Each rabbit in every group had unrestricted access to a specialized laboratory diet and drinking water. To ensure a methodical approach, the rabbits were divided into three main groups, each further subdivided into two sub-groups.

Groups

The rabbits were partitioned into groups A, B, and C. Group A served as the control group and received a placebo in the form of normal saline. Groups B and C were administered a daily dose of 30 ml/kg/day of 30% ethanol via a pediatric nasogastric tube. Moreover, group C underwent an additional treatment regimen, wherein they were provided with vitamin E at a dosage of 50 mg/kg/day, dissolved in 2 ml of distilled water, and also administered through a nasogastric tube [14,15]. Each of the three groups was further segmented into two subgroups, labeled as I and II, based on the duration of the experiment: one group underwent an eight-week experimental period, while the other experienced a four-week experimental duration. Subgroups undergoing the eight-week experiment were designated as "category E8," while those undergoing the four-week experiment were labeled as "category E4." This classification allowed for a more detailed analysis of the effects over varying time frames within each group.

At the end of the experiment, the animals were subjected to anesthesia using isoflurane inhalation. Cardiac perfusion was then carried out using normal saline and 4% paraformaldehyde. Subsequently, the entire liver was carefully dissected, extracted for further processing, and prepared for examination under a light microscope.

Tissue processing

The liver samples were sectioned and then immersed in 10% neutral buffered formalin for 24 hours to undergo fixation. After this initial fixation period, they were transferred to freshly prepared 10% neutral buffered formalin. A meticulous processing and embedding protocol in paraffin followed, with each liver sample being carefully handled to produce blocks suitable for further sectioning. Utilizing a microtome, tissue sections measuring 5 µm in thickness were meticulously crafted. These sections then underwent staining using eosin and hematoxylin (H&E) stain for subsequent analysis.

Data analysis

To conduct a microscopic examination, three slides were randomly selected from each specimen and observed under 4x, 10x, and 40x magnifications. Portal inflammation was detected and documented in the liver using the Knodel histological activity index [16]. To evaluate the stromal congestion, central vein size, and sinusoidal diameters were measured at 40X magnification using ImageJ Fiji software (U. S. National Institutes of Health, Bethesda, MA) at 10 random fields in each slide, following which the mean was calculated. Statistical comparisons among the groups were carried out employing the one-way ANOVA test, while analyses within each group were conducted using independent sample t-tests. All statistical analyses were performed using the statistical software Statistical Package for Social Sciences (SPSS), version 22.0 (IBM Corp., Armonk, NY), and a significance level of P < 0.05 was chosen to determine statistical significance.

## Results

**Figure 1:**
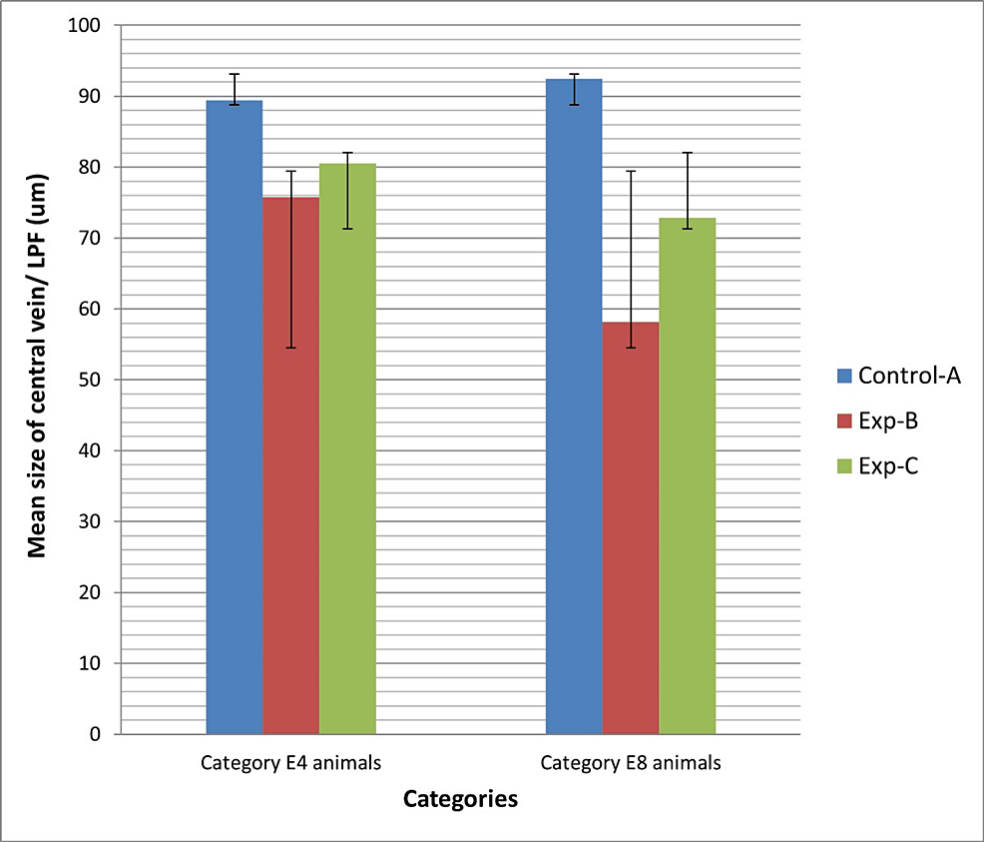
Means and standard deviations of central vein size of all groups in both category e4 and e8 animals.

**Figure 2:**
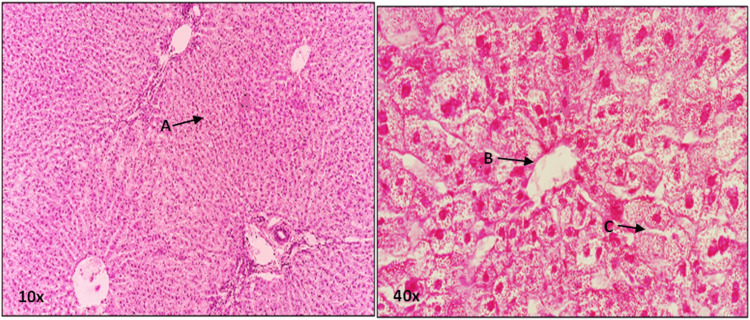
A photomicrograph depicting 5 µm thick H&E stained sections from group C-II, presented at 10X and 40X magnifications. At 10X magnification “A” and at 40x magnification “C” points to hepatic sinusoid congestion with decreased diameter. At 40X magnification, “B” points to compressed central vein. H&E: Hematoxylin and eosin.

Means and standard deviations of central vein size in all groups are shown in Figure 1. In category E4, a highly significant difference in central vein size was found among AII, BII, and CII groups, as well as between groups BII and CII (P values of 0.001 and 0.03, respectively). Similarly, in category E8, a highly significant difference in central vein size was observed among AI, BI, and CI groups, as well as between groups BI and CI (P values of 0.001 and 0.01, respectively). Notably, a significant difference in central vein size was observed between experimental groups B-I and B-II, with a P value of 0.025. Likewise, a significant difference in central vein size was evident in the comparison between experimental groups C-I and C-II, with a P value of 0.003. This demonstrates the reduction in central vein size over time in alcoholic liver injury, as depicted in Figure 2.

**Figure 3:**
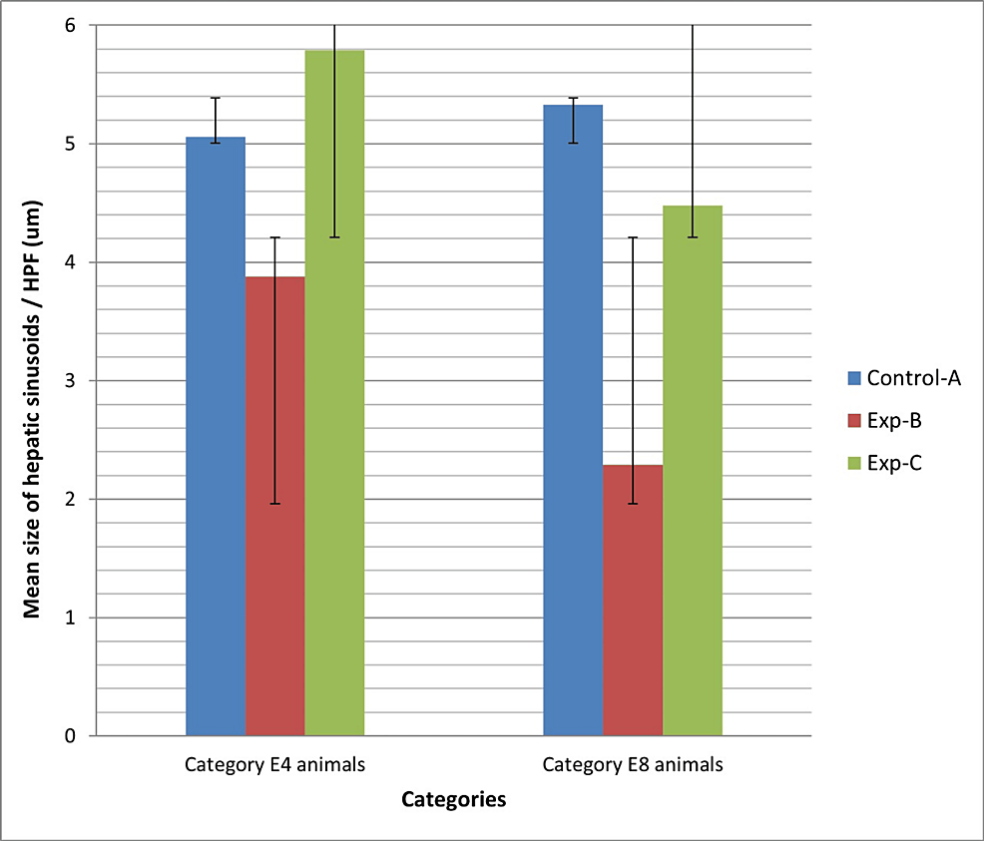
Means and standard deviations of the hepatic sinusoid size of all groups in both category e4 and e8 animals.

The means and standard deviations of hepatic sinusoid size across all groups are illustrated in Figure 3. Within category E4, a highly significant distinction in hepatic sinusoid size was identified among groups AII, BII, and CII, as well as between groups BII and CII (with respective P values of 0.004 and 0.034). Similarly, within category E8, a highly significant variation in central vein size was observed among groups AI, BI, and CI, as well as between groups BI and CI (with P values of 0.000 and 0.001, respectively). Notably, a significant difference in central vein size was also noted between experimental groups B-I and B-II, with a P value of 0.020. Similarly, a significant difference in central vein size was observed in the comparison between experimental groups C-I and C-II, with a P value of 0.003. These findings underscore the diminution in hepatic sinusoid size over time in the context of alcoholic liver injury, as depicted in Figure 2. 

**Figure 4:**
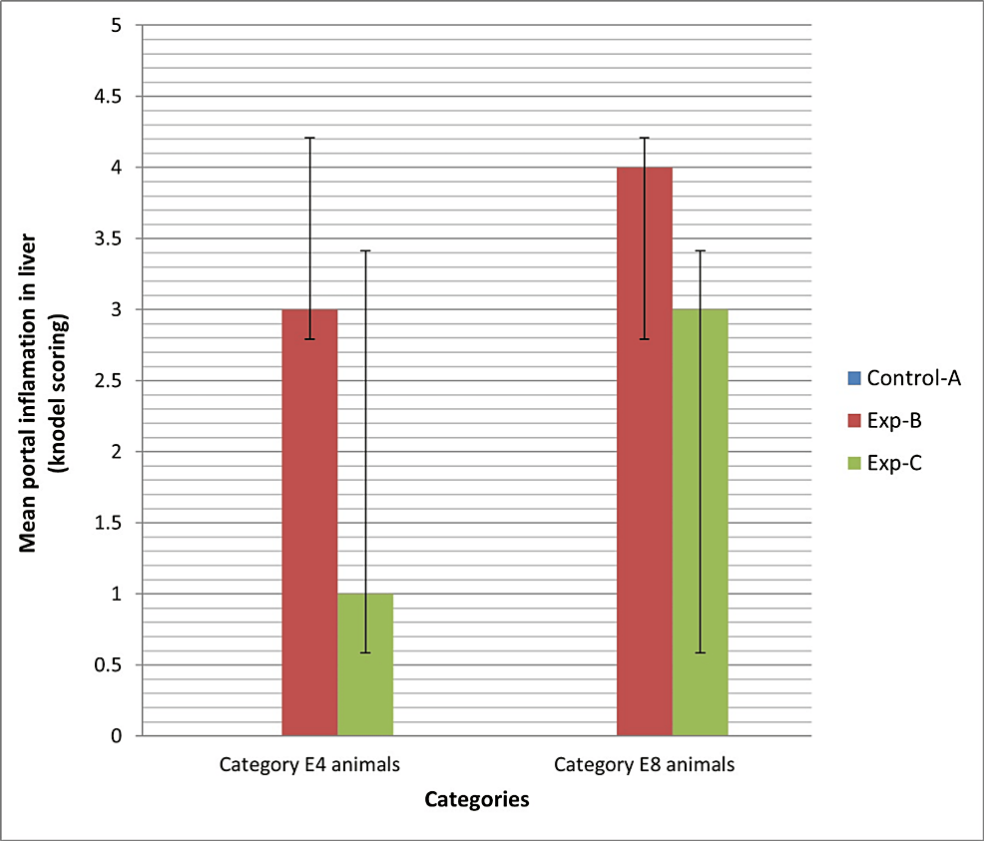
Means and standard deviations of portal inflammation Knodel scoring of all groups in both category e4 and e8 animals.

**Figure 5:**
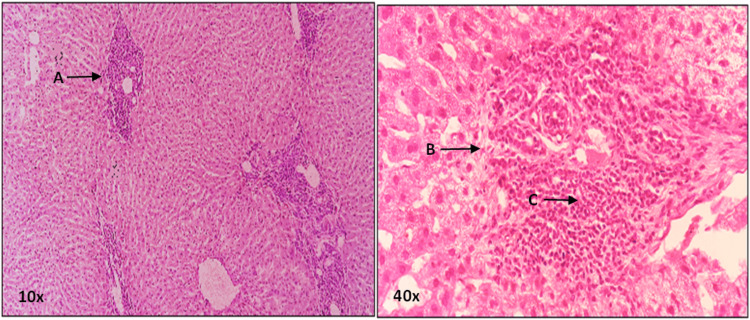
A photomicrograph depicting 5µm thick H&E stained sections from group C-II, presented at 10X and 40X magnifications. At 10X magnification “A” and at 40x magnification “B” points at portal triad, highly infiltrated by inflammatory cells. At 40X magnification, “C” points at inflammatory cells present in portal triad area. H&E: Hematoxylin and eosin.

Figure 4 illustrates the means and standard deviations of portal area invasion by inflammatory cells based on the Knodel Histological Activity Index across all groups. In category E4, a notable discrepancy in portal inflammatory cell infiltration was detected among AII, BII, and CII groups, as well as between groups BII and CII (P values of 0.02 and 0.04, respectively). Similarly, within category E8, a significant variance in portal inflammatory cell infiltration was noted among AI, BI, and CI groups, as well as between groups BI and CI (P values of 0.03 and 0.04, respectively). Importantly, a noteworthy difference in portal inflammatory cell infiltration was observed between experimental groups B-I and B-II, with a P value of 0.020. Likewise, substantial variance in portal inflammatory cell infiltration was evident in the comparison between experimental groups C-I and C-II, with a P value of 0.03. These findings show the effectiveness of vitamin E in limiting portal inflammatory cell infiltration in alcoholic liver injury, as delineated in Figure 5.

## Discussion

Alcoholic liver injury is a result of prolonged and excessive alcohol intake. Central to this condition is inflammation, characterized by the infiltration of inflammatory cells into the liver tissue. This inflammatory response initiates a cascade of liver damage, progressing through different stages. Initially, it manifests as fatty liver (steatosis), but with continued alcohol abuse, it can advance to more severe conditions such as alcoholic hepatitis and ultimately cirrhosis, wherein the liver becomes scarred and its function significantly impaired. Alcoholic liver disease (ALD) has a significant impact on liver blood flow, primarily manifesting as the progression of liver fibrosis and cirrhosis. These pathological states entail the deposition of fibrous tissue within the liver, which distorts its natural structure and functionality. As this fibrous tissue accumulates, it obstructs the flow of blood through the liver, resulting in heightened pressure within the organ's blood vessels. This elevation in pressure, termed portal hypertension, is a hallmark consequence of advanced ALD. Only male animals are used in the current research to avoid any hormonal changes that occur during the animal's estrous cycles that could affect the results [17].

The central vein and hepatic sinusoid sizes were smaller in the experimental groups compared to the control groups in both categories. Among these groups, animals in experimental group B, treated solely with alcohol, exhibited the smallest values for both parameters. This finding highlights the role of vitamin E in mitigating some of the harmful factors contributing to alcoholic liver injury to a certain degree. The results from our study align with those of another investigation, where researchers observed a reduction in central vein and sinusoidal diameter attributed to alcohol consumption, along with a negative relationship between hepatocyte size and sinusoidal dimensions [18]. In this study, we observed a clear infiltration of inflammatory cells in the portal areas of the experimental groups. Upon comparison, we found that the grade of inflammatory cell infiltration was higher in experimental group B compared to experimental group C in both category E4 and E8 animals. This finding suggests that vitamin E may be beneficial in reducing hepatic inflammation caused by alcohol. In this regard, our research findings align with those of a study that showcases the efficacy of vitamin E in mitigating the inflammatory response initiated by alcohol in the liver of mice [19]. This convergence underscores the consistent evidence supporting the beneficial effects of vitamin E in combating alcohol-induced liver inflammation. The results of the current study indicate that vitamin E supplementation shows potential as a therapeutic approach for ALD. The current study is consistent with another study suggesting that vitamin E can protect the liver from harmful changes caused by the ingestion of zinc oxide nanoparticles [20]. Nevertheless, additional research is necessary to clarify the ideal dosage, treatment duration, and criteria for selecting patients for vitamin E therapy. Additionally, it is crucial to account for potential interactions with other medications and underlying health conditions when managing ALD. Future investigations should also assess the effectiveness of combination therapies that target various pathways implicated in ALD development.

Limitations of the study include the small sample size, which may restrict the applicability to a wider population. Consequently, this limitation could undermine the study's statistical power and its capacity to identify significant effects. Furthermore, the study design-whether observational or a randomized controlled trial-poses inherent limitations. For example, observational studies may struggle to control for confounding variables, whereas randomized controlled trials may encounter challenges related to blinding or ensuring participant compliance.

## Conclusions

Vitamin E can present itself as a highly promising therapeutic remedy for alcoholic liver disease (ALD), thanks to its potent anti-inflammatory and antioxidant characteristics. Its ability to diminish the infiltration of inflammatory cells and alleviate stromal congestion suggests that it could effectively impede or potentially reverse the advancement of liver damage in individuals with ALD. Nevertheless, further investigation is imperative to comprehensively elucidate its mechanisms of action and refine its clinical application in addressing this multifaceted ailment.
